# Synthesis of Novel Polymeric Acrylate-Based Flame Retardants Containing Two Phosphorus Groups in Different Chemical Environments and Their Influence on the Flammability of Poly (Lactic Acid)

**DOI:** 10.3390/polym12040778

**Published:** 2020-04-01

**Authors:** Jacob Sag, Philipp Kukla, Daniela Goedderz, Hendrik Roch, Stephan Kabasci, Manfred Döring, Frank Schönberger

**Affiliations:** 1 Department of Polymer Synthesis, Fraunhofer Institute for Structural Durability and System Reliability LBF, Schlossgartenstraße 6, D-64289 Darmstadt, Germany; Jacob.Sag@lbf-extern.fraunhofer.de (J.S.); philipp.kukla@lbf.fraunhofer.de (P.K.); Daniela.Goedderz@lbf.fraunhofer.de (D.G.); manfred.doering@lbf.fraunhofer.de (M.D.); 2 Department of Bio-based Plastics, Fraunhofer Institute for Environmental, Safety and Energy Technology, UMSICHT, Osterfelder Straße 3, D-46047 Oberhausen, Germany; h.roch@cabka.com (H.R.); stephan.kabasci@umsicht.fraunhofer.de (S.K.)

**Keywords:** polylactic acid, phosphorus-based flame retardant, acrylate-based flame retardant

## Abstract

Novel polymeric acrylate-based flame retardants (FR 1–4) containing two phosphorus groups in different chemical environments were synthesized in three steps and characterized via nuclear magnetic resonance (NMR) spectroscopy, thermogravimetric analysis (TGA), differential scanning calorimetry (DSC), and mass spectrometry (MS). Polylactic acid (PLA) formulations with the synthesized compounds were investigated to evaluate the efficiency of these flame retardants and their mode of action by using TGA, UL94, and cone calorimetry. In order to compare the results a flame retardant polyester containing only one phosphorus group (ItaP) was also investigated in PLA regarding its flame inhibiting effect. Since the fire behavior depends not only on the mode of action of the flame retardants but also strongly on physical phenomena like melt dripping, the flame retardants were also incorporated into PLA with higher viscosity. In the UL94 vertical burning test setup, 10% of the novel flame retardants (FR 1–4) is sufficient to reach a V-0 rating in both PLA types, while a loading of 15% of ItaP is not enough to reach the same classification. Despite their different structure, TGA and cone calorimetry results confirmed a gas phase mechanism mainly responsible for the highly efficient flame retardancy for all compounds. Finally, cone calorimetry tests of the flame retardant PLA with two heat fluxes showed different flame inhibiting efficiencies for different fire scenarios.

## 1. Introduction

Due to oil scarcity and environmental issues, green alternatives of petrochemical products have gained a lot of impact in recent years. [1,2] One promising candidate for replacing traditional fossil-based polymer materials is poly (lactic acid) (PLA). It is not only derived from renewable resources, but also biodegradable. [3] Good physical properties and ease of fabrication allowed PLA to be applied in packages, drug delivery, and textiles. [4,5,6] Further potential applications in automotive and electrical industry would be possible, but are restricted because of its flammability. Therefore, flame retardants (FRs) such as phosphorus-containing compounds, nitrogen-containing compounds, and mineral fillers were incorporated and their effect on flame retardancy of PLA was investigated. [7,8,9,10,11] One of the most reported FRs among phosphorus-containing compounds are 9,10-dihydro-9-oxa-10-phosphaphenanthrene-10-oxide (DOPO) derivatives. Long et al. synthesized, characterized, and investigated various bridged DOPO derivatives (DOPO_2_-ethyl, DOPO_2_-phenethyl, and DOPO_2_-naphtalene) in PLA. [12,13,14] With a 10 wt.% addition of either one of these compounds PLA composites achieved a UL94 V-0 rating with decreased peak heat release rate (PHRR) values. [13] Liqiang et al. investigated the effect of three different amine-containing DOPO-based flame retardants on the flame resistance of PLA. Upon incorporation of 20 wt.% of one of these compounds a UL94 V-0 rating was obtained. [15] Senlong et al. reported that the flame retardant performance of PLA composites is enhanced by melt blending of neat PLA with DOPO containing PLA (P-PLA), which was synthesized by chain extension of L-Lactide with a DOPO derivative. [16] Flame retardancy tests indicated that the melt blended PLA with a loading of 10 wt.% of P-PLA reached a UL94 V-1 rating with a decreased PHHR value. Further research groups incorporated and investigated other DOPO derivatives in PLA, such as a DOPO-montmorillonite based compound, a DOPO-modified Co-based metal-organic framework (MOF) and a DOPO containing silesquioxane. [17,18,19] Besides these petrochemical flame retardants efforts were made to produce greener alternatives for flame retardant PLA involving the incorporation of biomass derived charring agents or flame retardants derived from biobased chemicals like pentaerythritol. [11] Lignin, starch, cellulose, cyclodextrin, and casein were incorporated in PLA and their flame retardant effect was evaluated. The PLA composites achieved a UL94 V-0 rating upon addition of 20–30 wt.% of one of these biomass derived compounds. [20,21,22,23,24,25] Xuan et al. synthesized melamine salts of bicyclic pentaerythritol phosphate alcohol (IFR-I and IFR-II). The incorporation of the new compounds in PLA improved the flame retardancy, reaching a V-0 classification with 20 wt.% addition in the best case. [26] Zhan et al. reported the synthesis and incorporation of spirocyclic pentaerythritol bisphosphorate diphosporyl melamine (SPDPM) in PLA. At a concentration of 25 wt.% SPDPM a V-0 rating was obtained. [8]

In this work, four novel flame retardants (FR 1–4) based on 2-hydroxyethyl acrylate (HEA) were synthesized and characterized by nuclear magnetic resonance (NMR) spectroscopy, thermogravimetric analysis (TGA), differential scanning calorimetry (DSC), and mass spectrometry (MS) measurements. Their flame inhibiting effect was then investigated in PLA. HEA itself is derived from acrylic acid and ethylene oxide, which both can industrially be produced from biorenewables. [27,28] PLA composites containing the new FRs were prepared via twin-screw extrusion. The flame retardancy was investigated from hot melt pressed samples by using TGA, UL94, and cone calorimetry. Since phosphorus-containing flame-retardants can promote action both in the gas phase and in the condensed phase, depending on the chemical environment of the phosphorus atom, [29] the synthesized FR 1–4 contain two different phosphorus groups. In order to evaluate the efficiency of the synthesized flame retardants another flame retardant (ItaP) bearing only one phosphorus group was also incorporated in PLA via twin-screw extrusion and investigated regarding its flame retardancy. ItaP is a biobased flame retardant polyester of the DOPO adduct of dimethyl itaconate and ethylene glycol, which already showed good flame retardant effects in various polyesters, but was not investigated in PLA yet. [30,31] 

## 2. Materials and Methods

Both PLA types (NatureWorks 3251D and 3052D) were obtained from Resinex (Zwingenberg, Germany). PLA 3251D has a Melt Flow Rate (MFR) of 80 (g/10 min, 210 °C, 2.16 kg) and a melting point of 160 °C. PLA 3052D has a MFR of 14 (g/10 min, 210 °C, 2.16 kg) and a melting point of 150 °C. ItaP was provided by Chemische Fabrik Budenheim KG (Budenheim, Germany). Unless stated otherwise, solvents and chemicals were obtained from VWR (Radnor, PA, USA) and used without further purification. DOPO was supplied by Metadynea Austria GmbH (Krems, Austria).

### 2.1. General Procedure

All synthesis procedures were carried out under nitrogen atmosphere and dry solvents were used. Nuclear magnetic resonance (NMR) data were obtained on a Bruker NanoBay 300 spectrometer (Bruker, Ettlingen, Germany). Chemical shifts are reported as δ values relative to the solvent peak in ppm. Trimethylsilane was used as an internal standard. Thermodesorption-MS experiments were performed using a Finnigan MAT 95 with constant heating rate of 25 K min^−1^ in the temperature range of 40–400 °C. TGA was carried out using a TGA Q500 from TA Instruments, Inc. (New Castle, DE, USA) with a heating rate of 10 K min^−1^ under constant nitrogen atmosphere.

A corotating twin-screw extruder (Thermo Scientific Process 11 from Fisher Scientific (Schwerte, Germany), screw diameter: 11 mm) with a screw rotation speed of 200 rpm was used for compounding. The first temperature zone was set at 190 °C, all other temperature zones (2–7) were set at 200 °C. The melt strand was cooled in a water bath and granulated in a pelletizing system (VariCut Granulator, ThermoFischer, Schwerte, Germany).

UL94 samples were obtained by hot pressing (Collin) of the compounds at 200 °C and 60 bar for 5 min. UL94 vertical burning test was done in a WAZAU burning chamber according to DIN IEC 60,695-11-10 using a 50 W burner flame.

The cone calorimeter tests were carried out according to ISO 5660 on a cone calorimeter from Fire Testing Technology (East Grinstead, England) at a heat flux of 35 and 50 kW/m^2^ on square specimen (100 mm × 100 mm × 3 mm). 

### 2.2. Synthesis

#### 2.2.1. Synthesis of the Acrylates 1 a–d

In a three-necked round-bottom glass flask with magnetic stirring 0.5 mol (1.0 eq.) hydroxyethyl acrylate (HEA) and 0.5 mol (1.0 eq.) *N*-methylimidazole were dissolved in 250 mL dichloromethane (for 1 a acetonitrile was used) and cooled to 0–5 °C in an ice bath. In case of the acrylates 1 a–c 0.25 mol (0.5 eq.) of a dichloride containing phosphorus (V) compound was added dropwise into the flask. For acrylate 1 d 0.17 mol (0.33 eq.) phosphoryl chloride was added. The temperature was kept at 0–5 °C during the addition and after removing the ice bath the mixture was stirred for 4 h at room temperature. The formed methylimidazole hydrochloride was dissolved by adding water (200 mL) to the reaction mixture. The organic phase was separated, washed with water twice and subsequently dried over anhydrous magnesium sulfate. The acrylates 1 a–d were isolated (yields: 85–95%) by removing the dichloromethane using a rotary evaporator. 

1 a: ^1^H-NMR (CDCl_3_, 300 MHz, rt): δ [ppm] = 6.46 (dd, 2 H, *J* = 17.3; 1.4 Hz); 6.14 (dd, 2 H, *J* = 17.3; 10.4 Hz); 5.90 (dd, 2 H, *J* = 10.4; 1.4 Hz); 4.67 (dd, 2 H, *J* = 11.9; 2.4 Hz); 4.49–4.24 (m, 10 H); 4.14 (d, 2 H, *J* = 11.9 Hz); 4.03 (dd, 2 H, J = 11.9; 2.4 Hz). ^31^P-NMR (CDCl_3_, 122 MHz, rt): δ [ppm] = −7.76. APCI-MS [m/z]: 457.07 [C_15_H_22_O_12_P_2_ + H]^+^.

1 b: ^1^H-NMR (CDCl_3_, 300 MHz, rt): δ [ppm] = 7.43–7.14 (m, 5 H); 6.45 (dd, 2 H, *J* = 17.3; 1.4 Hz); 6.13 (dd, 2 H, *J* = 17.3; 10.4 Hz); 5.88 (dd, 2 H, *J* = 10.4; 1.4 Hz); 4.46–4.34 (m, 8 H). ^31^P-NMR (CDCl_3_, 122 MHz, rt): δ [ppm] = −6.63. APCI-MS [m/z]: 371.09 [C_16_H_19_O_8_P + H]^+^.

1 c: ^1^H-NMR (CDCl_3_, 300 MHz, rt): δ [ppm] = 7.77–7.59 (m, 2 H); 7.51–7.28 (m, 3 H); 6.26 (dd, 2 H, *J* = 17.3; 1.5 Hz); 5.95 (dd, 2 H, *J* = 17.3; 10.4 Hz); 5.73 (dd, 2 H, *J* = 10.4; 1.5 Hz); 4.32–4.07 (m, 8 H). ^31^P-NMR (CDCl_3_, 122 MHz, rt): δ [ppm] = 19.75. APCI-MS [m/z]: 355.10 [C_16_H_19_O_7_P + H]^+^.

1 d: ^1^H-NMR (CDCl_3_, 300 MHz, rt): δ [ppm] = 6.40 (dd, 3 H, *J* = 17.3; 1.4 Hz); 6.09 (dd, 3 H, *J* = 17.3; 10.4 Hz); 5.83 (dd, 3 H, *J* = 10.4; 1.4 Hz); 4.41–4.16 (m, 12 H). ^31^P-NMR: (CDCl_3_, 122 MHz, rt): δ [ppm] = −1.39. APCI-MS [m/z]: 393.10 [C_15_H_21_O_10_P + H]^+^.

#### 2.2.2. Synthesis of the Monomers 2 a–d

In a three-necked round-bottom glass flask with magnetic stirring 0.4 mol (1.0 eq.) acrylate 1 a–d and 0.06 mol trimethylamine (0.15 eq.) were dissolved in 500 mL toluene and heated to 90 °C. For the synthesis of monomer 2 a–c 0.46 mol (1.15 eq.) DOPO was added in portions. For the synthesis of monomer 2 d 0.86 mol (2.15 eq.) DOPO were added in portions. After 4 h at 90 °C the solvent was evaporated and the residue was dissolved in dichloromethane. The organic phase was washed with water twice and subsequently dried over anhydrous magnesium sulfate. The monomers 2 a–d were obtained after removing the dichloromethane by rotary evaporation (yields: 90–95%).

2 a: ^1^H-NMR (CDCl_3_, 300 MHz, rt): δ [ppm] = 8.04–7.82 (m, 3 H); 7.78–7.62 (m, 1 H); 7.60–7.46 (m, 1H); 7.45–7.34 (m, 1 H); 7.33–7.13 (m, 2 H); 6.46 (ddd, 1 H, *J* = 17.3; 7.7; 1.4 Hz); 6.14 (ddd, 1 H, *J* = 17.3; 10.4; 7.7); 5.89 (ddd, 1 H, *J* = 11.9; 10.4; 1.4 Hz); 4.77–4.51 (m, 2 H); 4.50–3.92 (m, 14 H); 2.82–2.57 (m, 2 H); 2.49–2.24 (m, 2 H). ^31^P-NMR (CDCl_3_, 122 MHz, rt): δ [ppm] = 36.19; −7.62. APCI-MS [m/z]: 673.10 [C_27_H_31_O_14_P_3_ + H]^+^.

2 b: ^1^H-NMR (CDCl_3_, 300 MHz, rt): δ [ppm] = 8.09–7.83 (m, 3 H); 7.80–7.67 (m, 1 H); 7.61–7.49 (m, 1 H); 7.47–7.07 (m, 8 H) 6.45 (ddd, 1 H, *J* = 17.3; 4.2; 1.4 Hz); 6.12 (ddd, 1 H, *J* = 17.3; 10.4; 4.6 Hz); 5.89 (ddd, 1 H, *J* = 10.4; 3.8; 1.4 Hz); 4.48–4.35 (m, 4 H); 4.34–4.18 (m, 4 H); 2.78–2.57 (m, 2 H); 2.49–2.31 (m, 2 H). ^31^P-NMR (CDCl_3_, 122 MHz, rt): δ [ppm] = 35.92; −6.53. APCI-MS [m/z]: 587.12 [C_28_H_28_O_10_P_2_ + H]^+^.

2 c: ^1^H-NMR (CDCl_3_, 300 MHz, rt): δ [ppm] = 8.09–7.55 (m, 6 H); 7.66–7.35 (m, 5 H); 7.35–7.11 (m, 3 H); 6.42 (ddd, 1 H, *J* = 17.3; 4.2; 1.4 Hz); 6.10 (ddd, 1 H, *J* = 17.3; 10.4; 4.2 Hz); 5.86 (ddd, 1 H, *J* = 10,4; 3,3; 1,4 Hz); 4.51–4.07(m, 8 H); 2.78–2.49 (m, 2 H); 2.49–2.23 (m, 2 H). ^31^P-NMR (CDCl_3_, 122 MHz, rt): δ [ppm] = 35.99; 20.02. APCI-MS [m/z]: 571.12 [C_28_H_28_O_9_P_2_ + H]^+^.

2 d: ^1^H-NMR (CDCl_3_, 300 MHz, rt): δ [ppm] = 8.03–7.85 (m, 6 H); 7.80–7.68 (m, 2 H); 7.61–7.49 (m, 2 H); 7.46–7.35 (m, 2 H); 7.35–7.07 (m, 4 H); 6.47 (dtd, 1 H, *J* = 17.3, 3.8, 1.5 Hz, 1 H); 6.25–6.05 (m, 1 H); 5.89 (dtd, 1 H, *J* = 10.4, 3.8, 1.5 Hz); 4.53–3.97 (m, 12 H); 2.84–2.59 (m, 4 H); 2.53–2.29 (m, 4 H). ^31^P-NMR (CDCl_3_, 122 MHz, rt): δ [ppm] = 35.99, −1.20. APCI-MS [m/z]: 825,16 [C_39_H_39_O_14_P_3_ + H]^+^.

#### 2.2.3. Synthesis of Polymeric Flame Retardants (FRs) 1–4

In a three-necked round-bottom glass flask with magnetic stirring 0.5 mol (1.0 eq.) monomer 2 a–d was solved in 500 mL toluene and heated to 95 °C. A solution of 0.01 mol (0.02 eq.) AIBN in 50 mL toluene (0.2 M) was added and the mixture was stirred for 4 h. After decanting the solvent and drying the residue in vacuo at 90 °C the flame retardants 1–4 are obtained (yields: 95–98%). 

FR 1: Insoluble in any solvents.

FR 2: ^1^H-NMR (CDCl_3_, 300 MHz, rt): δ [ppm] = 8.04–7.80 (m, 3 H); 7.78–7.62 (m, 1 H); 7.59–7.43 (m, 1 H); 7.43–7.00 (m, 8 H); 4.63–3.89 (m, 9 H); 2.78–2.48 (m, 2 H); 2.48–2.22 (m, 2 H); 2.16–1.34 (m, 3 H). ^31^P-NMR (CDCl_3_, 122 MHz, rt): δ [ppm] = 36.45; −6.52.

FR 3: ^1^H-NMR (CDCl_3_, 300 MHz, rt): δ [ppm] = 8.02–7.60 (m, 6 H); 7.60–7.32 (m, 5 H); 7.32–7.13 (m, 2 H); 4.42–4.01 (m, 8 H); 2.74–2.44 (m, 2 H); 2.44–2.16 (m, 2 H); 1.96–1.30 (m, 3 H). ^31^P-NMR (CDCl_3_, 122 MHz, rt): δ [ppm] = 36.26; 20.18.

FR 4: ^1^H-NMR (CDCl_3_, 300 MHz, rt): δ [ppm] = 8.08–7.80 (m, 6 H); 7.80–7.62 (m, 2 H), 7.62–7.44 (m, 2 H); 7.44–7.06 (m, 6 H); 4.44–3.98 (m, 12 H); 2.86–2.52 (m, 4 H); 2.52–2.23 (m, 4 H); 2.10–1.37 (m, 3 H). ^31^P-NMR (CDCl_3_, 122 MHz, rt): δ [ppm] = 35.98; −1.12.

## 3. Results and Discussion

### 3.1. Synthesis and Characterization of the Flame Retardants

**Figure 1:**
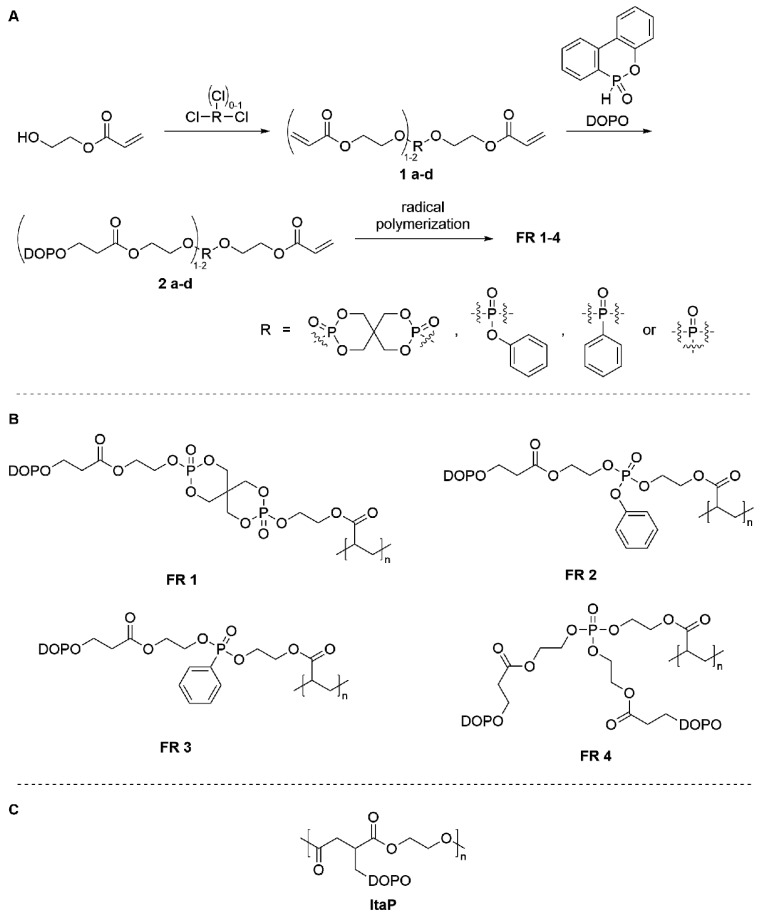
Synthesis route to flame retardant (FR) 1–4 (**A**), full structure of FR 1–4 (**B**) and structure of a flame retardant polyester containing only one phosphorus group (ItaP) (**C**).

The three-step synthetic route for the FRs 1–4 is illustrated in Figure 1. The monomers were synthesized in high yields (85–95%) by phosphorylation of HEA with a phosphorus containing di- or trichloro-compound followed by Phospha-Michael addition with DOPO. For the first step *N*-methylimidazole is used as acid scavenger, since usage of triethylamine leads to a small amount of unidentified impurities. A slight excess of DOPO is used to favor that, after radical polymerization of these monomers 2 a–d, the obtained FRs 1–4 show thermoplastic properties. This excess of DOPO reacts with the double bonds of the acryloyl groups and therefore reduces the amount of crosslinking during polymerization. Due to better blending while extruding thermoplastic properties are favored. 

**Figure 2:**
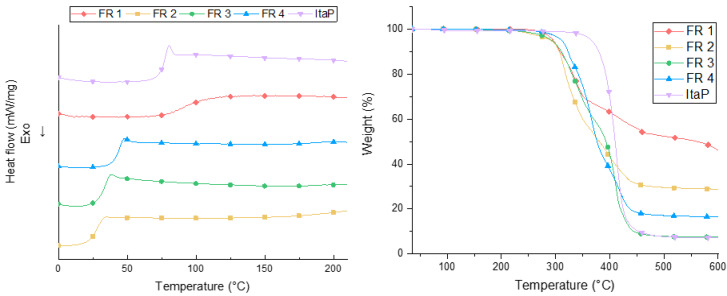
Differential scanning calorimetry (DSC) (left) and thermogravimetric analysis (TGA) (right) curves of FR 1–4 and ItaP with a heating rate of 10 K/min from 0–210 °C (DSC) and 35–600 °C (TGA) in nitrogen atmosphere.

**Figure 3:**
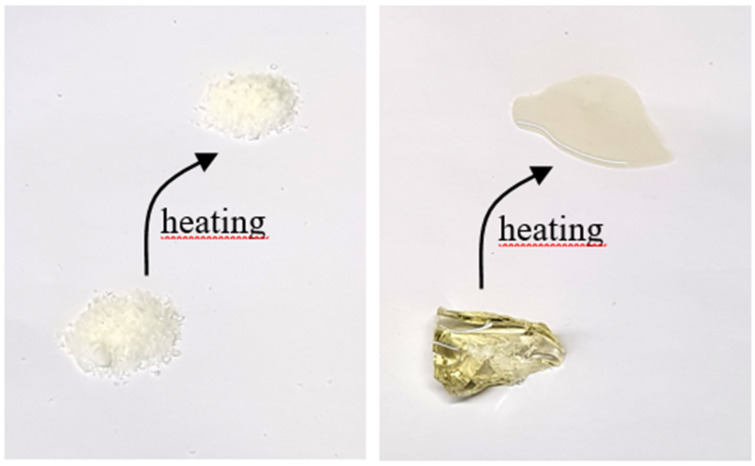
FR 1 (left) and FR 2 (right) before (bottom) and after (top) heating. FR 3 and FR 4 behave under heat like FR 2.

The chemical structures of the intermediates were confirmed by ^1^H-, ^31^P-NMR-, and atmospheric-pressure chemical ionization mass spectrometry (APCI-MS). After polymerization, FRs 2–4 show no residual double bonds in ^1^H-NMR spectroscopy indicating the successful formation of polymers. FR 1 could not be characterized by NMR due to its insolubility in common solvents. The thermal properties of the polymers 1–4 were investigated by differential scanning calorimetry (DSC) and thermogravimetric analysis (TGA) (Figure 2). Table 1 summarizes the thermal properties containing the glass transition temperature (T_g_), the initial degradation temperature (T_1%_) at which 1% of the mass is lost, and the residue at 600 °C. Figure 2 shows DSC curves for the synthesized flame retardants. The glass transition temperature of the synthesized compounds ranges from 28 to 91 °C. Although no melting point can be observed in the DSC curves, FRs 2–4 gradually soften upon heating, which is shown in Figure 3. This behavior is typically observed for amorphous thermoplastics.

The TGA results show decomposition temperatures (T_1%_) between 230 and 269 °C for the FRs 1–4 (Figure 2, Table 1). ItaP has the highest thermal stability with a T_1%_ of 330 °C. Considering simpler compounds with the same backbone, [32,33] this difference in thermal stability of the phosphorus containing analogs is not surprising. A varying amount of residue at 600 °C implies different mode of actions for the FRs 1–4. Flame retardant 3 and ItaP have the lowest residues of only 7–8% at 600 °C indicating mainly gas phase activity. FR 1, 2, and 4, which all have at least one phosphate group, show significantly higher residues and thus indicate a condensed phase activity alongside their gas phase activity induced by their DOPO moieties.

The thermal properties of the polymeric flame retardants tend to pronounced condensed phase activity in the following order: FR 1 > FR 2 > FR 4 > FR 3 = ItaP which correlates with the oxygen content in the chemical environment of the phosphorus atoms. 

**Figure 4:**
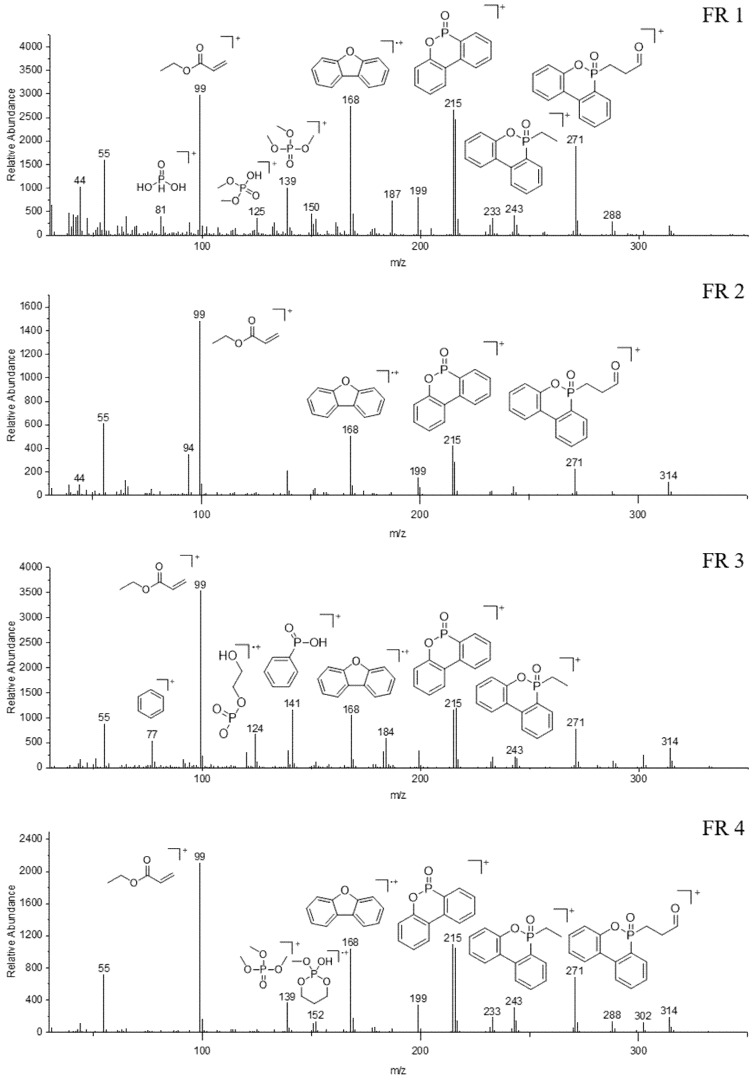
Mass spectra of FRs 1–4.

Mass spectrometry was performed to further elucidate the mode of action. Figure 4 shows the detected pyrolysis fragments. All compounds have a main fragment of m/z = 99 u in common which can be assigned to the acrylate. Fragments with m/z = 168 u, 215 u, 243 u, and 271 u originate from the DOPO decomposition, whereas different phosphorus fragments can be found additionally for each flame retardant 1–4. The fragmentation pattern of spiro alkylphosphonates is well described in the literature and can be applied on the spiro phosphate of FR 1. [34] Therefore, it is obvious that the decomposition of FR 1 leads to lots of very small fragments in comparison to FR 2–4.

### 3.2. Thermal Analysis of Flame Retardant Formulations

**Figure 5:**
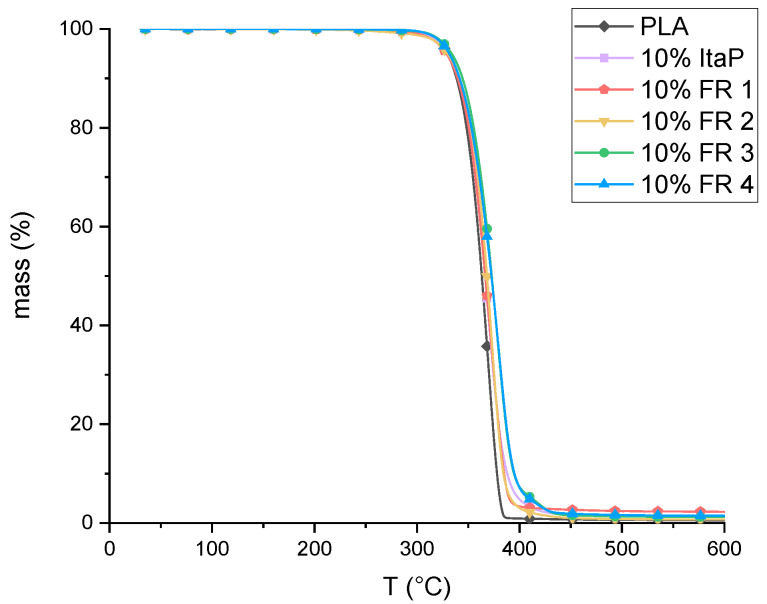
TGA curves of the flame retardant formulations with a heating rate of 10 K/min in nitrogen atmosphere.

The influence of incorporation of FRs 1–4 and ItaP on the decomposition of PLA was investigated using TGA. The curves of the flame retardant formulations in PLA under nitrogen atmosphere are shown in Figure 5. The related thermal decomposition data of the temperature at 1% weight loss (T_1%_) and the residual yield at 600 °C obtained from the curves are summarized in Table 2. It can clearly be observed that incorporation of 10 wt.% of the flame retardants does not alter the decomposition mechanism of PLA nor lead to a char formation upon decomposition. While this was expected for FR 3 and ItaP due to a lack of phosphate moieties, it is surprising at first glance, that FR 1, 2, and 4 do not form char despite having phosphate groups in their structure and showing higher residues when decomposing in bulk (Table 1). Then again, it is already reported that the release of phosphorus fragments in a phosphorus containing flame retardant is highly influenced by the polymer matrix, the phosphorus species and the concentration of phosphorus in the specimen. [35] Even though phosphate-based flame retardants are reported to act in the condensed phase by dehydration, it is already proven that if phosphate esters are released into the gas phase rather than react with the decomposing polymer, they still show high flame inhibiting effects. [29,36] 

### 3.3. Burning Behavior of the Flame Retardants in PLA

UL94 tests were performed in order to investigate the flame retardant effect of the synthesized compounds. ItaP, a DOPO-containing polyester which already showed good fire behavior but wasn’t investigated in PLA yet, was also tested in order to compare the efficiency of the synthesized flame retardants. [30,31] Since the fire behavior depends not only on the mode of action of the flame retardants and their interaction with the polymeric matrix, but also strongly on physical phenomena like melt dripping which correlates to the viscosity of the melt, two PLAs with different melt viscosities are investigated. [37] The composition of the formulations and the results are represented in Table 3 for the PLA with low viscosity (MFR, g/10 min, 210 °C, 2.16 kg: 80) and Table 4 for the PLA with higher viscosity (MFR, g/10 min, 210 °C, 2.16 kg: 14). The low viscosity PLA reached V-2 classification in the UL94 test due to rapid melt dripping of the specimen flowing away from the flame during testing. Incorporation of one of the flame retardants always reduced the burning time t_1_/t_2_, but not the classification in every case. V-0 rating was obtained for the PLA with 10% of any of the flame retardants 1–4. In case of FR 3 even 5% were sufficient to reach this classification. 

For comparison, addition of 15% ItaP only achieved V-2 rating. Various research groups already showed that overlap of the decomposition temperatures of the flame retardant and the polymer are essential for a good flame inhibiting effect, [38,39] making ItaP in general a good candidate for PLA (Table 1 and Table 2). Even though the decomposition temperatures of ItaP and PLA are closer to each other, compared to FR 1–4, ItaP got the worse classification in the UL94 test. Since melt dripping of the specimen is so fast, the sample isn’t reaching temperatures high enough for ItaP to be effective enough. FRs 1–4 decompose earlier and therefore show better results.

FR 1, 2, and 4 contain phosphorous atoms in the same chemical environment and neither TGA results of the formulation nor the UL94 results of the PLA composites differ. Therefore, only FR 4 was selected for further analysis as a model containing both phosphate and phosphinate moieties. For comparison FR 3 consisting of phosphonate and phosphinate moieties as well as the phosphinate containing ItaP were also further tested. The neat PLA with higher viscosity burned down completely since melt dripping of the specimen was not fast enough to escape the flame. Similar to the lower viscous PLA, incorporation of flame retardants result in shorter burning times. A V-0 rating was obtained for PLA with 10% FR 3, and 10% FR 4, being again more efficient than ItaP, that still reaches only V-2 classification even with 15%.

**Figure 6:**
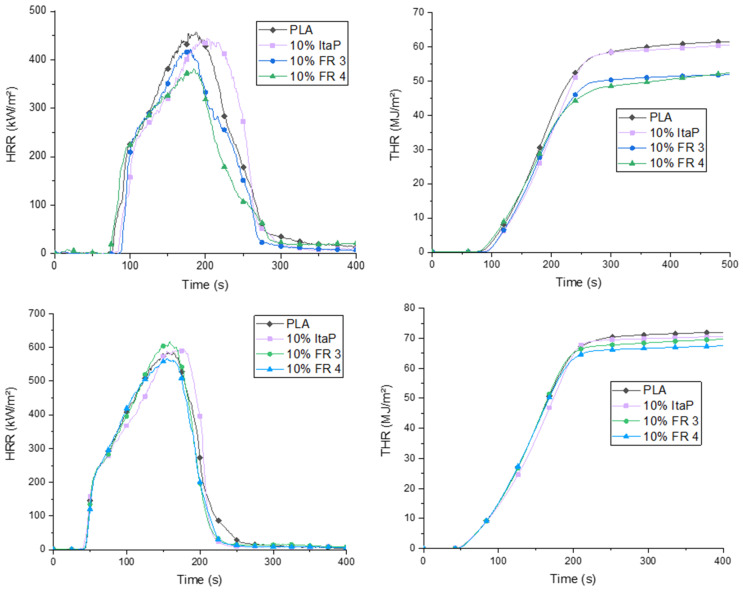
Heat release rate (HRR) (left) and total heat release (THR) (right) curves for flame retardant formulations at a heat flux of 35 kW/m^2^ (top) and 50 kW/m^2^ (bottom).

Cone calorimetry tests were performed for the low viscosity PLA formulations containing 10% flame retardant at a heat flux of 35 and 50 kW/m^2^ to assess performance of the flame retardants for different fire scenarios. The results are listed in Table 5. Heat release rate (HRR) and total heat release (THR) curves are compared in Figure 6. In general, the addition of the flame retardants 3 and 4 in PLA induced only a slight reduction in peak heat release rate (PHRR) values when irradiated with 35 kW/m^2^ with FR 4 being the more efficient one. Addition of ItaP lead to no changes in the PHRR. Regarding the time till PHRR is reached (t_max_), ItaP shifted it to higher values meaning a slower flame propagation in a fire scenario while FR 3 has the opposite effect and reduces t_max_ slightly. Compared to neat PLA FR 4 has no influence on t_max_. THR values decreased clearly for the formulation with 10% FR 3 and FR 4 being again more efficient than ItaP. The total smoke production (TSP) is increased for all formulations containing flame retardants, which is a result of incomplete combustion in the gas phase and therefore, [40] confirming the gas phase mechanism of these compounds. The time to ignition (TTI) values shortened for the formulations containing FR 4 and prolonged for the FR 3 and ItaP composite.

When irradiated with 50 kW/m^2^, the effect of the incorporated flame retardants is almost neglectable with FR 4 having the largest influence by slightly reducing PHRR and THR values. These results indicate that the used flame retardants are not suited for slowing down a fire once it is fully developed. Considering all results from both the burning tests, the synthesized flame retardants show excellent results in the UL94 test and rather mediocre performance in the cone calorimetry test. Since these tests represent different fire scenarios, there is only an indirect correlation between these methods, therefore, rendering the results unsurprising. [40]

## 4. Conclusions

In this work, four novel phosphorus-based flame retardants (FR 1–4) derived from HEA were synthesized and characterized by NMR, TGA, and MS. The flame retardants 1–4 were incorporated in two PLAs with different melt viscosities in various loadings. For comparison, ItaP, a DOPO containing polyester, which already showed good flame inhibition in various polymers, but was not incorporated in PLA yet, was also tested. In the UL94 test the synthesized flame retardants showed excellent flame inhibiting properties resulting in a V-0 rating upon addition of only 10 wt.%. In the case of ItaP, 15 wt.% was still not sufficient for reaching V-0. The corresponding TGA results indicate that the flame retardants 1–4 and ItaP act mainly through a gas-phase mechanism. Cone Calorimetry showed only small effects on PHRR and THR and confirmed the gas-phase action due to higher TSP values. This work demonstrates a new class of highly efficient flame retardants for PLA, which will be further investigated in ongoing studies regarding a synergistic effect with flame retardants being active in the condensed phase as well as their flame retardancy in other various polymeric materials.

## Figures and Tables

**Table 1:** Tg, T_1%_ and Residue of FR 1–4 and ItaP obtained by DSC and TGA with a heating rate of 10 K min^−1^ in nitrogen atmosphere.

FR	T_g_ (°C)	T_1%_ (°C)	Residue at 600 °C (wt.%)
1	91	269	50
2	28	231	29
3	32	233	8
4	43	265	17
ItaP	80	330	7

**Table 2:** Thermal Properties of the Flame Retardant Formulations.

Composition	T_1%_ (°C)	Calculated Residue at 600 °C (wt.%)	Determined Residue at 600 °C (wt.%)
PLA	317	-	0.5
10% ItaP	315	0.7	1.3
10% FR 1	310	5.0	2.3
10% FR 2	305	2.9	0.7
10% FR 3	320	0.8	1.2
10% FR 4	318	1.7	1.5

**Table 3:** UL94 classification of the PLA composites with low viscosity.

			Composition (%)				UL94 (1.6 mm)	
Sample	PLA	ItaP	FR 1	FR 2	FR 3	FR 4	t1/t2 (s)	Rating
**PLA1**	100	-	-	-	-	-	3/1	V-2
**PLA2**	95	5	-	-	-	-	0/0	V-2
**PLA3**	95	-	5	-	-	-	0/0	V-2
**PLA4**	95	-	-	5	-	-	0/0	V-2
**PLA5**	95	-	-	-	5	-	0/0	V-0
**PLA6**	95	-	-	-	-	5	0/0	V-2
**PLA7**	90	10	-	-	-	-	0/1	V-2
**PLA8**	90	-	10	-	-	-	0/0	V-0
**PLA9**	90	-	-	10	-	-	0/0	V-0
**PLA10**	90	-	-	-	10	-	0/0	V-0
**PLA11**	90	-	-	-	-	10	0/0	V-0
**PLA12**	85	15	-	-	-	-	0/0	V-2

**Table 4:** UL94 Classification of the PLA composites with higher viscosity.

	Composition (%)				UL94 (1.6 mm)	
Sample	PLA	ItaP	FR 3	FR 4	t1/t2 (s)	Rating
**PLA13**	100	-	-	-	78/0	n.c.
**PLA14**	95	-	5	-	2/0	V-2
**PLA15**	95	-	-	5	0/0	V-2
**PLA16**	90	10	-	-	2/3	V-2
**PLA17**	90	-	10	-	0/0	V-0
**PLA18**	90	-	-	10	0/0	V-0
**PLA19**	85	15	-	-	1/0	V-2

**Table 5:** Cone Calorimetry results of flame retardant formulations in PLA with a sample thickness of 3 mm.

	Composition	TTI (s)	PHRR (kw/m^2^)	t_max_ (s)	THR (MJ/m^2^)	TSP (m^2^)
35 kW/m^2^	PLA	80	457	188	62.1	0.1
	10% ItaP	86	444	202	61.2	3.9
	10% FR 3	88	422	178	52.1	3.3
	10% FR 4	74	381	185	53.9	3.0
50 kW/m^2^	PLA	41	583	164	72.5	0.01
	10% ItaP	38	590	157	71.9	5.3
	10% FR 3	40	617	160	71.1	4.7
	10% FR 4	41	567	156	68.5	5.0
